# Induced Osteogenesis in Plants Decellularized Scaffolds

**DOI:** 10.1038/s41598-019-56651-0

**Published:** 2019-12-27

**Authors:** Jennifer Lee, Hyerin Jung, Narae Park, Sung-Hwan Park, Ji Hyeon Ju

**Affiliations:** 1 Divison of Rheumatology, Department of Internal Medicine, College of Medicine, Seoul St. Mary’s Hospital, The Catholic University of Korea, 222 Banpo-daero, Seocho-gu, Seoul 06281 Republic of Korea; 2 CiSTEM Laboratory, Convergent Research Consortium for Immunologic Disease, College of Medicine, Seoul St. Mary’s Hospital, The Catholic University of Korea, 222 Banpo-daero, Seocho-gu, Seoul 06591 Republic of Korea; 3 Catholic iPSC Research Center, College of Medicine, The Catholic University of Korea, Seoul, 137-701 Republic of Korea

**Keywords:** Induced pluripotent stem cells, Stem-cell differentiation

## Abstract

A three-dimensional (3D) culture system that closely replicates the *in vivo* microenvironment of calcifying osteoid is essential for *in vitro* cultivation of bone-like material. In this regard, the 3D cellulose constructs of plants may well serve as scaffolds to promote growth and differentiation of osteoblasts in culture. Our aim in this study was to generate bone-like tissue by seeding pluripotent stem cells (hiPSCs), stimulated to differentiate as osteoblasts in culture, onto the decellularised scaffolds of various plants. We then assessed expression levels of pertinent cellular markers and degrees of calcium-specific staining to gauge technical success. Apple scaffolding bearing regular pores of 300 μm seemed to provide the best construct. The bone-like tissue thus generated was implantable in a rat calvarial defect model where if helped form calcified tissue. Depending on the regularity and sizing of scaffold pores, this approach readily facilitates production of mineralized bone.

## Introduction

It is well established that three-dimensional (3D), rather than two-dimensional (2D), culture offers a microenvironment closer to *in vivo* conditions, better enabling *in vitro* development of organoids. Similar with other organs, there is a growing clinical need for bone organoids, which may be particularly suitable substitutes for less available autologous bone grafts, helping to repair critical bony injuries or congenital defects. Unfortunately, current engineering techniques involving bone are largely restricted to 3D culture of osteoblasts. Hence, the term ‘bone-like tissue’ seems more apt than ‘bone organoid’. With respect to *in vitro* genesis of bone-like tissue, the use of scaffolds in 3D cultures has become a major investigative technique^1^.

The range of inherent properties is an important aspect of any scaffolding biomaterial, which should be biocompatible, easily manipulated, and structurally sound, providing proper mechanical support and bioactivity. To this end, various natural or synthetic materials have been utilised as biomaterials in scaffold development. Specifically, nanofibers of electrospun synthetic polymer^2^ and composite hydroxyl apatite (HA)^3^ or collagen^4^ scaffolds have been devised for 3D culture of osteoblasts. Natural chitosan-based fibres have also been used to culture osteoblasts^5^. Although many publications have expounded on optimal 3D culture conditions for bone-like tissue development, the ideal properties and structure have yet to be fully clarified. There are numerous materials and methods still under investigation^6^.

Induced pluripotent stem cells (iPSCs) are generated by reprogramming the transduction of four genes (*OCT3/4, SOX2, C-MYC*, and *KLF4*)^7^, known as Yamanaka factors. Such cells have become promising tools in disease modelling and regenerative medicine, given their potential to replicate any cell and the corresponding tissue. iPSCs may be produced from peripheral blood mononuclear cells^8^, thus affording easy and virtually unlimited access to tissues of interest, without invasive biopsies.

In an earlier effort, decellularised apple was used as a porous cellulose backbone for 3D culture of mammalian cells^9^. The authors subsequently indicated that this plant-derived cellulose scaffold was biocompatible when subcutaneously implanted in the backs of mice^10^. Similarly, a cellulose-based scaffold has been applied for culturing and osteoblastic differentiation of human mesenchymal stem cells^11^. These successes prompted our use of this natural, readily available, and easily handled scaffolding in the 3D engineering of bone. We were thus compelled to determine whether iPSCs could be cultured and differentiate as osteoblasts within a plant-derived cellulose scaffold. We also explored the prospective clinical merits, using resultant bone organoid as *in vivo* implants.

## Results

### Decellularised apple provides cellulose scaffold for 3D cell cultures of hiPSCs

**Figure 1:**
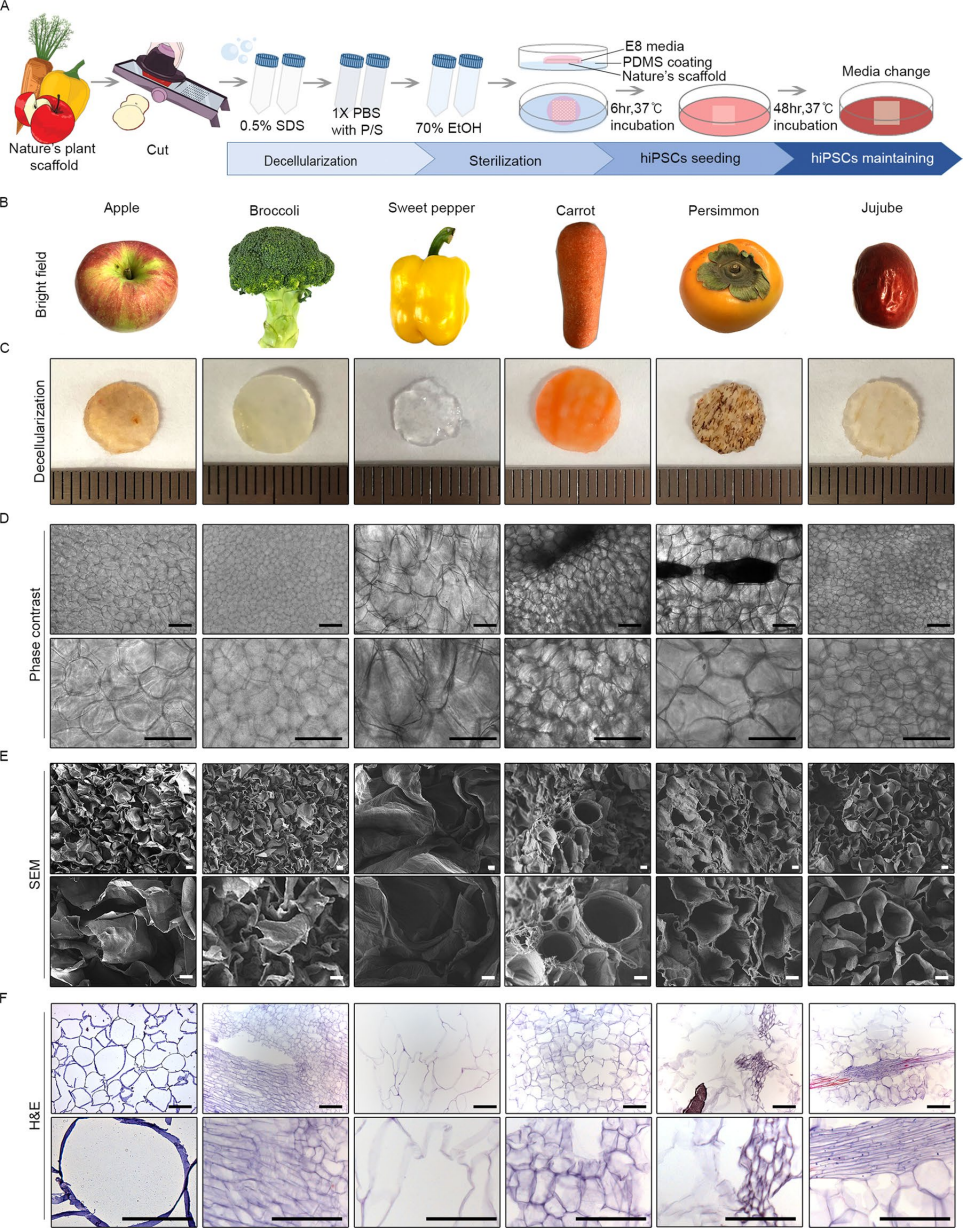
Decellularised plants provide cellulose-based scaffolds with pores of various sizes and shapes: (**A**) Schematic of strategy to develop decellularised plant scaffolds, seeding induced human pluripotent cells onto scaffolds for incubation; (**B**) Images of plants under investigation; (**C**) Images of plants after decellularisation (**D**) Phase contrast images of scaffolds (original magnifications: 100x and 200x); (**E**) Scanning electron microscopic images of scaffolds (original magnifications: 200x and 500x); and (**F**) Haematoxylin & eosin-stained images of scaffolds (original magnifications: 200x and 400x). Scale bars: 10 μm (H&E) and 100 μm.

**Figure 2:**
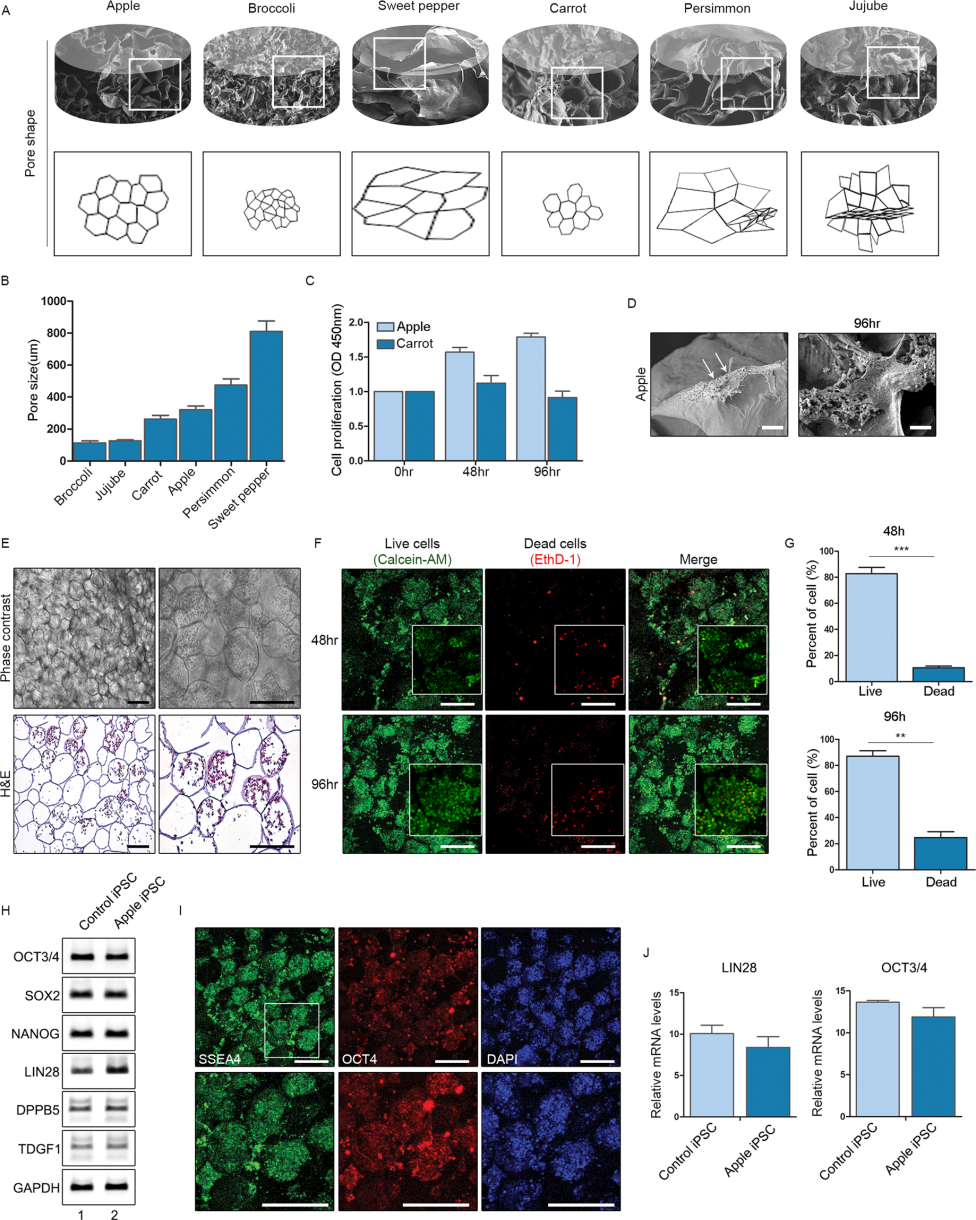
Induced pluripotent stem cells (iPSCs) cultivated in apple-derived scaffolds: (**A**) Drawings of various plant scaffolds showing shape and pore sizes; (**B**–**D**) Pore sizes, cell proliferation assay (CCK-8), and scanning electron microscopic images of human induced pluripotent stem cells (hiPSCs) seeded in apple scaffolds; (**E**) Phase contrast (top) and haematoxylin & eosin-stained (bottom) images of hiPSCs seeded onto apple scaffolds (original magnifications: 100x and 200x); (**F**) Views of live and dead cells in cultures after 48 h (top panels) and 96 h (bottom panels); (**G**) Proportion of the live and dead cells in cultures after 48 h and 96 h; (**H**) mRNA expression levels of iPSC markers in control iPSCs and iPSCs cultured in apple scaffolding; (**I**) Immunofluorescence staining of iPSC markers in iPSCs cultured in apple scaffolding; and (**J**) mRNA expression levels of *LIN28* and *OCT3/4* in control iPSCs and iPSCs cultured in apple scaffolding (*p < 0.01; **p < 0.005; and ***p < 0.001).

We first decellularised various plants (apple, broccoli, sweet pepper, carrot, persimmon, and jujube) to create porous cellulose scaffolds, as previously described^9^ (Fig. 1A–C). Briefly, sliced apple (0.5 mm thick) was cut into pieces (1 × 1 cm) for sequential immersion in 0.5% sodium dodecyl sulphate (SDS) solution (to decellularise) and 70% ethanol (to sterilise). The remaining cellulose constructs harboured pores of various shapes and sizes (Fig. 1D–F). In addition to apples, which have proven useful for successful culturing of cell lines, we also tested carrot and persimmon, both being similar to apple in pore shape and size (Fig. 2A,B). Once seeded with hiPSCs, only cells cultivated in apple scaffolding survived, as confirmed by Cell Counting Kit-8 (CCK-8) assay after 96 h (Fig. 2C) and by scanning electron microscopy (Fig. 2D). In the different type of scaffolds, cells maintained their poorly spread and did not proliferate well (Fig. 2C, Supplementary Fig. S3**)**. Viable hiPSCs were also confirmed within apple scaffolding under phase contrast microscopy and in haematoxylin and eosin (H&E)-stained histological preparations (Fig. 2E). To gauge cell viability and proliferative capacity, we performed LIVE/DEAD analysis. Cellular proliferation within apple scaffolding increased at both 48 h and 96 h, whereas numbers of dead cells did not (Fig. 2F,G). Cells surviving in culture after 96 h still expressed stem cell markers, (OCT3/4, SOX2, NANOG, LIN28, DPPB5, TDGF1, and SSEA4) at levels comparable to iPSCs cultured in 2D media (Fig. 2H,J), implying retention of pluripotency by hiPSCs within scaffolds.

### Osteoblastic differentiation of hiPSCs within apple scaffolding

**Figure 3:**
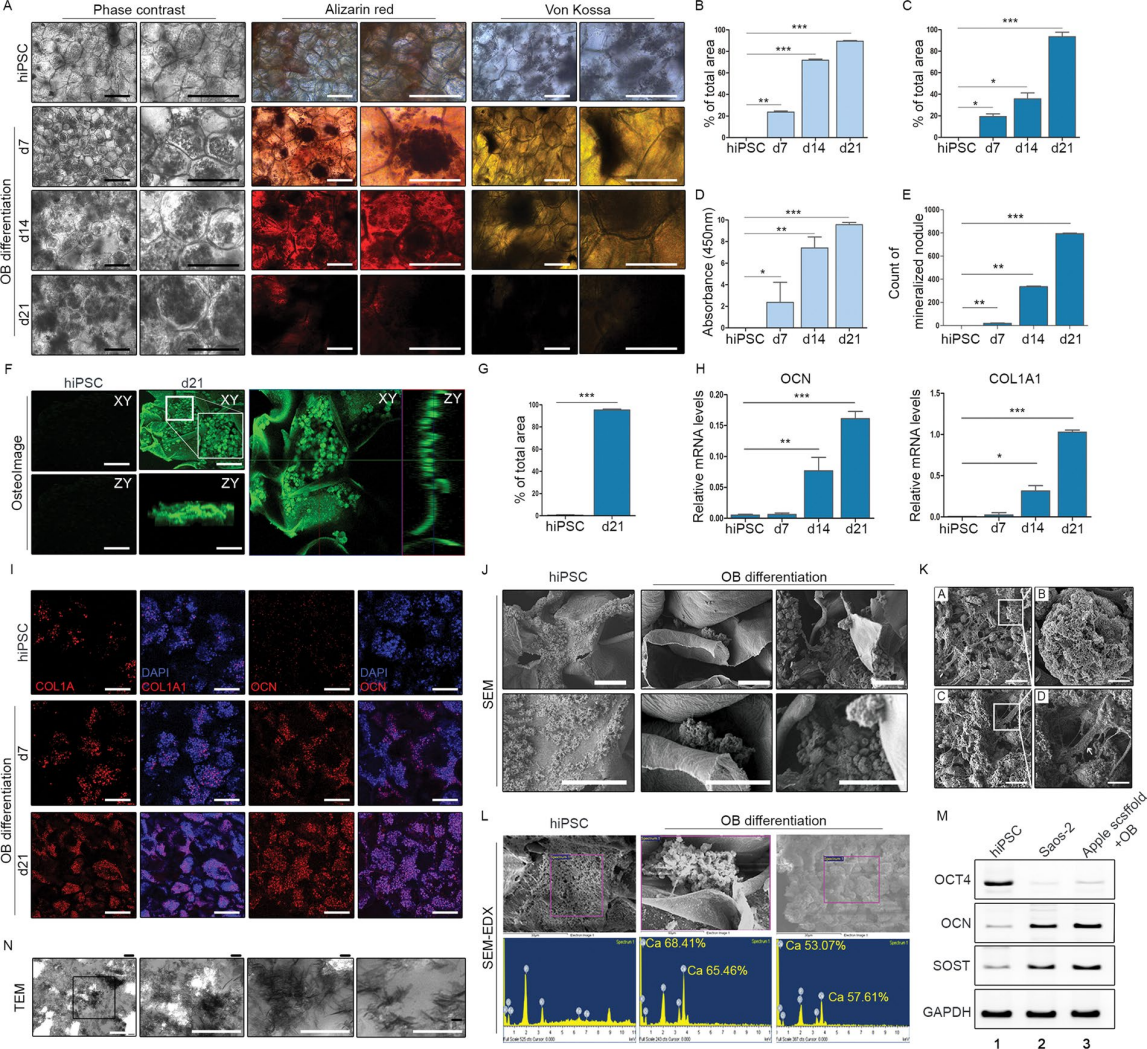
Osteoblastic differentiation of human induced pluripotent stem cells (hiPSCs) within 3D scaffolds: (**A**) Phase contrast images, Alizarin Red S stain, and von Kossa stain of hiPSCs before and after osteoblastic differentiation; (**B**–**E**) Alizarin Red S and von Kossa staining of hiPSCs before and after osteoblastic differentiation (proportions of positively stained areas, absorbance at 450 nm, and counts of mineralised nodules shown in graphs); (**F**,**G**) Osteoimaging of hiPSCs before and after osteoblastic differentiation; (**H**) Levels of osteocalcin and type I collagen mRNA expressed by hiPSCs before and after osteoblastic differentiation (*p < 0.01; **p < 0.005; and ***p < 0.001); (**I**) Immunofluorescence staining of type I collagen and osteocalcin in hiPSCs and osteoblasts (nuclei stained via DAPI). Scale bars: 20 μm; (**J**,**K**) Scanning electron microscopic images after osteoblastic differentiation; (**L**) Energy dispersion X-ray spectrometer ranges obtained by focusing beam on mineralising nodules; (**M**) Expression levels of *OCT4*, *OCN*, and *SOST* mRNA after osteoblastic differentiation. Note: Full-length electrophoretic bands are presented in Supplementary Fig. 4. (**N**) Osteoblastic differentiation shown by transmission electron microscopy.

We then successfully cultured scaffolds seeded with hiPSCs for 21 days in osteogenic differentiation media. Phase contrast images showed mineralising nodules (Fig. 3A), confirmed by Alizarin Red S (Fig. 3A,B,D) and von Kossa (Fig. 3A,C,E) stains and by osteoimaging (Fig. 3F,G); and we consistently recorded high levels of mRNA expressed in time-dependent manner by osteogenic markers, such as osteocalcin (OCN) and type I collagen (COL-1) (Fig. 3H). Immunofluorescence stains also regularly showed increases in OCN and COL-1 expression levels, compared with hiPSCs at baseline (Fig. 3I). Transmission (Fig. 3N) and scanning (Fig. 3J,K) electron microscopy more clearly depicted the mineralisation within pores. Through energy-dispersive X-ray spectrometry (EDX), such fortification was readily linked to osteogenic differentiation (Fig. 3L). Of note, expression of sclerostin (SOST, an osteocyte marker) was evident in osteoblast (OB)-laden scaffolding, attesting to the osteocytic nature of these differentiated cells (Fig. 3M). We were able to mould the decellularised apple scaffolding in any manner, without loss of shape at any time when subjected to lifting by forceps (Supplementary Fig. S1). In assessing osteoblast-bearing apple scaffolds by confocal laser microscopy, ‘Apple scaffold + OB’ exceeded ‘Apple scaffold only’ or ‘Apple scaffold + hiPSCs’ in thickness and degree of surface roughness (Supplementary Fig. S2).

### Bone organoid implant promotes *in vivo* repair in rat calvarial defect model

**Figure 4:**
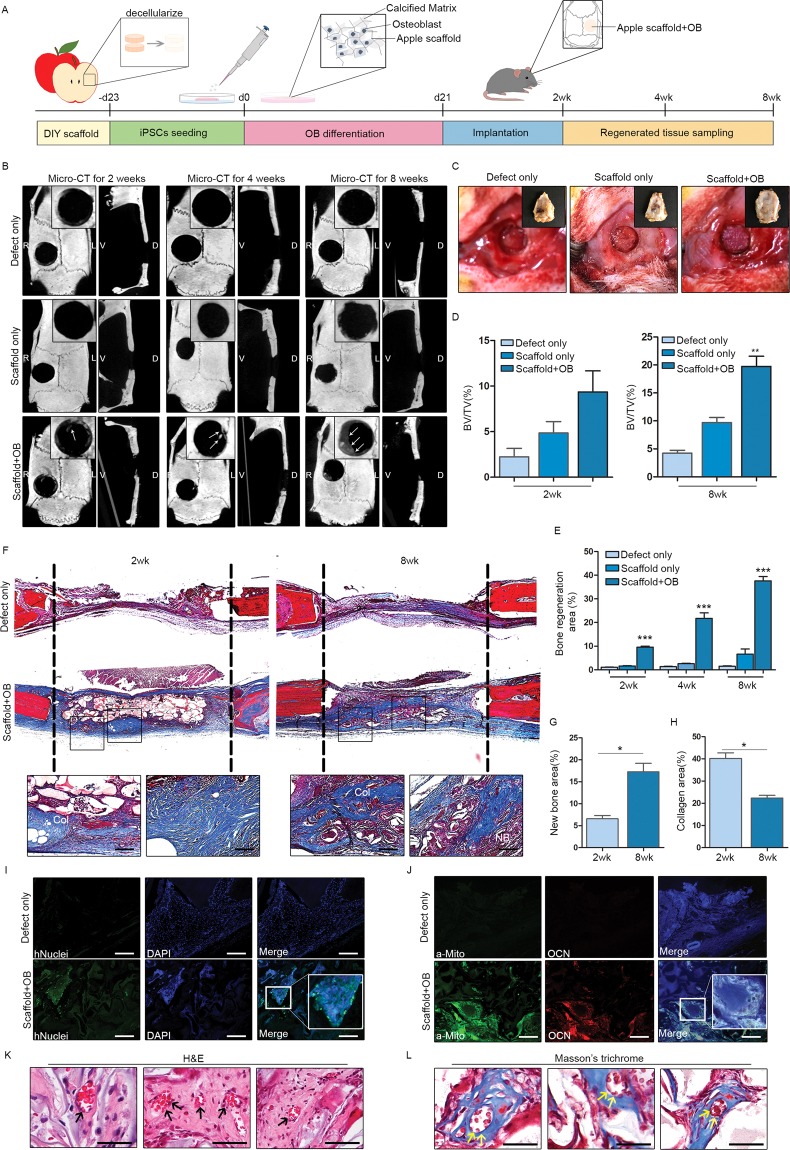
Bone-like graft implants in rat calvarial defect model to aid bone regeneration: (**A**) Schematic of strategy for bone-like graft development and implantation; (**B**) Microcomputed tomography (CT) views of calvarial defect sites at 2 weeks (left), 4 weeks (middle), and 8 weeks (right) after grafting, with representative images of defect only (top panels), scaffold only (middle panels), and osteoblast-laden scaffold implants (bottom panels); (**C**) Photos of calvarial defects without implant (left), scaffold only (middle), and osteoblast-laden scaffold implantation (right); (**D**) Proportion of bone volume (BV) to total volume (TV) 2 weeks (left) and 8 weeks (right) after implantation (*p < 0.01; **p < 0.005; and ***p < 0.001); (**E**) Proportion of bone regeneration 2 weeks (left), 4 weeks (middle), and 8 weeks (right) after implantation; (**F**) Masson’s trichrome stain of defect sites at 2 weeks and 8 weeks after implantation; (**G**,**H**) Proportion of new bone area and area of type I collagen deposition 2 weeks and 8 weeks after implantation; (**I**) Immunofluorescence staining of human cellular nuclei in graft, 8 weeks after implantation; (**J**) Immunofluorescence staining of human mitochondria and osteocalcin in graft, 8 weeks after implantation; and (**K**,**L**) Blood vessel formation in graft (H&E and Masson’s trichrome stains).

Finally, we explored the potential use of bone-like tissue elements as *in vivo* grafts, engaging a commonly used rat calvarial defect model to assess bone regeneration^12^. This entailed making two identical 5-mm circular defects in rat skulls and grafting only one to compare the rates of healing. After receiving cultured scaffolds as implants, the rats were sacrificed at 2, 4, and 8 weeks for serial evaluations (Fig. 4A). Microcomputed tomography (CT) and visual inspection at 8 weeks showed partial regeneration of implanted areas, growing from margins inward (Fig. 4B–D), with no signs of inflammation. Histologically, bone organoids appeared successfully engrafted, with type I collagen deposition by Masson’s trichrome stain (Fig. 4E–H). Because scaffolds themselves could conceivably encourage bone regeneration in rats, we screened implanted scaffolds for human cells, verifying the presence of human nuclei, mitochondria^13,14^, and osteocalcin by immunofluorescence staining (Fig. 4I,J) and indicating survival of hiPSC-derived osteoblasts *in vivo*. Moreover, we observed blood vessel-like structures within scaffolds that presumptively grew into the implant (Fig. 4K,L). Given the collective results, it is apparent that these bone organoid implants promote healing of defects *in vivo* and have capacity to provide grafted bone with needed blood supply.

## Discussion

The 3D culture system implemented herein relies on a plant-derived cellulose scaffold to optimize support of developing bone-like structures and ultimately permit *in vivo* implantation. It is also apparent that the grafted end-products promote bone regeneration. As a biomaterial, an apple-derived construct has several advantages, including xeno-free plant-derived properties, biocompatibility, easy handling, and low cost.

Although osteoblasts may be effectively cultured by 2D methods, 3D cultures have added benefits. In one study, expression levels of COL-1, osteocalcin, and osteonectin were found to be higher in hiPSC-derived osteoblasts generated via polyethersulfone nanofibrous 3D scaffold^2^. More recently, an effort to transform mouse iPSCs into osteoblasts yielded higher expression levels of LBSP (an osteoblast marker), as well as DMP1 and SOST (osteocyte markers), using atelocollagen as scaffolding rather than 2D culture media^15^.

By design, 3D culture systems should mimic the *in vivo* microstructures of various organs, ideally incorporating extracellular matrix (ECM) of targeted organs as scaffolding. Recently, this approach has been broadly attempted in organoids of liver^16,17^, thymus^18^, intestine^19^, and pancreas^20^, seeding cells onto native ECM after decellularisation. However, a donor organ is required for this purpose; and with respect to bone, autologous grafts (the best option for seeding) are not only limited in supply but also impose donor site problems. Instead, scaffolds are being fabricated from or coated with major components of bone ECM, such as HA or type I collagen, to generate bone-like tissue *in vitro*^21–23^. Furthermore, 3D bioprinting with cell-loaded bioink is emerging as another attractive option^24^, albeit rooted in high-tech devices. Despite the absence of bony ECM in our cellulose scaffolds, the resultant mineralised nodules were still well developed. In future endeavours, addition of ECM components to our system may be considered to perhaps enhance the osteogenic potential.

It is unclear why apple-derived scaffolds alone proved suitable for culturing of cells, whereas other candidate plants completely failed. Their ECM constituents are likely shared, so it is the unique mechanical properties of apple that ostensibly make a difference. Scaffolding for regenerative bone should be structurally interconnected and highly porous (≥90%), with adequately sized openings (≥100 μm) to allow cellular proliferation and vascular ingrowth, carrying nutrients and oxygen^5^. The pores of apple scaffolding, each~200 μm in diameter, are therefore amenable.

The openness of apple scaffolding also seems particularly conducive to vascular ingrowth, which is critical for successful implantation. Oxygenation and supplied nutrients are so important that co-seeding of endothelial cells and hiPSC-derived mesenchymal stem cells (MSCs) into calcium phosphate cement scaffolds has been investigated as a means of integrating vessel-like structures into bone organoids^25^. However, our system simply encouraged vascular ingrowth at implant sites, similar to a previous report documenting vascularization of implanted apple-derived cellulose scaffolds *in vivo*^9^. Furthermore, monocytes (as precursors of osteoclasts) may be delivered to bone organoids by this route, and white blood cells recruited via blood vessels may differentiate into osteoclasts, bolstering physiologic bony structures.

In clinical applications, biodegradability is an important aspect of scaffolds. After 8 weeks, our plant-derived scaffolding implants remained intact, showing no signs of degradation or immune response. Longer term observation is rightly needed to address the issue of degradability, but in any clinical application of iPSCs, there are usually concerns of related teratomas or malignancy. We found no evidence of teratoma formation in implanted defects. We also confirmed that the blood vessels originated from without, formed by endothelial cells of rat origin.

Our culture system is easy to reproduce and manipulate, and may be readily utilized without expensive devices or complicated techniques. Although the plant-derived scaffold itself may not be useful in regenerative medicine, it is nonetheless instructive on a fundamental level, underscoring that optimal genesis of bone-like tissue in 3D cultures is reliant on pore size and shape of the scaffolding material.

## Methods

### Ethical approval

All procedures involving animals were performed in accordance with the Laboratory Animals Welfare Act, the Guide for the Care and Use of Laboratory Animals, and Guidelines and Policies for Rodent Experimentation adopted by the Institutional Animal Care and Use Committee of the School of Medicine at the Catholic University of Korea; and the Institutional Review Board approved our study protocol (KC14TISI0334). We obtained hiPSCs from the Catholic iPSC Research Center, as detailed in a previous study^26^. IACUC and Department of Laboratory Animal (DOLA) in Catholic University of Korea, Songeui campus accredited the Korea Excellence Animal Laboratory Facility from Korea Food and Drug Administration in 2017 and acquired AAALAC International full accreditation in 2018.

### Preparation of natural plant scaffold

The plant scaffold was serially sliced at a uniform thickness (0.5 ± 0.3 mm) and cut to uniform lengths (0.9 ± 0.1 mm each). We measured the thickness and length of each plant scaffold with callipers. Plant tissue was decellularised in accord with a previously published protocol^9,10^, adding fruit samples to sterile 2.5-ml tubes with 0.5% SDS (Sigma-Aldrich, St Louis, MO, USA) solution and shaking at 180 RPM for 48 h. After washing three times with 1× phosphate-buffered saline (PBS) and 70% ethanol two times. 1× PBS with 1% penicillin/streptomycin (HyClone) and 1% amphotericin B (Sigma-Aldrich, St Louis, MO, USA), samples were incubated for 6 h. After washing three times with 1× phosphate-buffered saline (PBS) and incubate for 14 hours in E8 media.

### Visualization of natural plant-scaffolding structure

We examined pore sizes via electron and light microscopy (Olympus, Shinjuku, Tokyo, Japan). To visualize the porosity of natural plant scaffolds, H&E-stained histological preparations were used. Sections were cut at 5 μm, then deparaffinized and rehydrated for 10-min staining in Harris’s haematoxylin solution. Excess haematoxylin was removed by immersion in 1% HCl in 70% (v/v) ethyl alcohol for 5 s, followed by bluing in 0.5% ammonium hydroxide solution, Eosin Y (1%, w/v) counterstaining for 1.5 min, and a 1-min wash in running tap water. Sections were mounted on clear glass slides and coverslipped.

### Scanning electron microscopy (SEM)

We examined cell attachments and pore sizes of plant scaffolds via SEM. Plant scaffolds were washed three times with Dulbecco’s PBS (DPBS) and fixed in 2.5% glutaraldehyde (Duk San, Gyeongnam, South Korea). A gold coating was applied to observe the surface features by SEM (JSM-5600LV; JEOL, Akishima, Tokyo, Japan).

### *In vitro* culture of hiPSCs within natural plant scaffolds

Natural plant scaffolds were incubated in 24-well tissue culture plates. Each well was coated with polydimethylsiloxane (PDMS), prepared as a 1:10 mixture of hardener and elastomer (Sylgard 184; Dow Corning, Midland, MI, USA). The PDMS mixture was dispensed into each well, cured at 80 °C for 2 h, cooled at room temperature (RT), and washed with 1× PBS. Such coating is intended to prevent cell adhesion by creating a hydrophobic surface. A plant support was placed in each well of a PDMS-coated plate for seeding with 1 × 10^6^ hiPSCs suspended in 20 μl E8 medium. Cells were attached to plant scaffolds for 6 h in an incubator. The hiPSCs grown on PDMS-coated plates were maintained in E8 medium.

### *In vitro* osteogenesis by hiPSCs seeded onto natural plant scaffolds

Osteogenic differentiation medium (ODM) was prepared by adding 15% foetal bovine serum (FBS), 100 nM dexamethasone, 10 mM β-glycerol phosphate, and 50 μg/ml ascorbic acid to Dulbecco’s Modified Eagle Medium-Low glucose (DMEM-LG). Medium was replaced every 2 days in cultures incubated for 3 weeks. When hiPSCs seeded onto plant scaffolds were 80% confluent, we switched to ODM.

### Alizarin Red S and von kossa staining

Alizarin Red S staining served to confirm osteoblastic differentiation. To gauge degrees of calcium deposition, we washed differentiated plant scaffolds with sterile 1× PBS on Days 0, 7, 14, and 21, adding Alizarin Red S solution for 30 min. Non-specifically stained portions were rinsed several times with sterilised water. Stained areas were examined by light microscope (DMi8; Leica, Wetzlar, Germany). To quantify Alizarin Red staining, we added 10% (w/v) cetylpyridinium chloride solution at RT, allowing a 30-min reaction. Absorbance of the supernatant was recorded at 595 nm by microplate reader. A von Kossa stain was also performed to verify calcific deposits. After washing with PBS, the cells were fixed in 4% paraformaldehyde (PFA) for 20 min. After again washing with tap water, we treated samples with 5% silver nitrate solution, exposed them to ultraviolet (UV) light for 40 min, and then washed with tap water. Finally, samples were incubated with 5% sodium thiosulfate for 5 min and washed with tap water. Stained sections were viewed by light microscope^27,28^.

### Real-time polymerase chain reaction (PCR)

Real-time polymerase chain reaction and quantitative PCR (qPCR) were performed to assess expression levels of pluripotent and osteoblast-specific marker genes in osteoblast-differentiated scaffolds. We extracted the total RNA of these plant scaffolds using Trizol Reagent (15596026; Invitrogen [ThermoFisher], Carlsbad, CA, USA), using the RevertAid First Strand cDNA Synthesis Kit (K1621; ThermoFisher Scientific, Waltham, MA, USA) as directed for cDNA synthesis. A list of primers is provided in Table 1.

**Table 1:** PCR primer sequences used for RT-PCR analysis.

PCR product	Sequence (5′-3′)	Fragment size (bp)
OCT3/4	F-ACCCCTGGTGCCGTGAA	190
	R-GGCTGAATACCTTCCCAAATA	
SOX2	F-CAGCGCATGGACAGTTAC	321
	R-GGAGTGGGAGGAAGAGGT	
NANOG	F-AAAGGCAAACAACCCACT	270
	R-GCTATTCTTCGGCCAGTT	
LIN28	F-GTTCGGCTTCCTGTCCAT	122
	R-CTGCCTCACCCTCCTTCA	
DPPB5	F-CGGCTGCTGAAAGCCATTTT	215
	R-AGTTTGAGCATCCCTCGCTC	
TDGF1	F-TCCTTCTACGGACGGAACTG	140
	R-AGAAATGCCTGAGGAAAGCA	
GAPDH	F-GAATGGGCAGCCGTTAGGAA	414
	R-GACTCCACGACGTACTCAGC	

RT-PCR: reverse transcription polymerase chain reaction; F: Forward primer; R: reverse primer.

### Cell viability assay

Cell viability was based on two-color fluorescence analysis, using the LIVE/DEAD Viability/Cytotoxicity Assay Kit (L3224; Molecular Probes [ThermoFisher], Eugene, OR, USA) as directed by manufacturer. To confirm proliferation of hiPSCs seeded onto natural plant scaffolds, we used the Cell Counting Kit-8 assay (CCK-8; CK04-13, Dojindo Molecular Technologies, Inc), transferring supernatant after 48 h and 96 h to 96-well plates and adding 10 μl CCK-8 solution to each well. Absorbance was recorded at 595 nm by microplate reader.

### Cellular immunostaining

Pluripotency markers served to characterize cells, which were washed with 1× PBS and fixed by adding 4% PFA for 10 min at RT. We then added 0.1% Triton X-100 and incubated at RT for 10 min. Piercing of cell walls allowed the solution to penetrate. Thereafter, we added PBS buffer solution containing 2% bovine serum albumin (BSA) for 40 min. Anti-OCT4 antibody (ab19857; Abcam, Cambridge, UK) and anti-Stage-Specific Embryonic Antigen-4 (SSEA-4) antibody (MAB4304; Millipore Sigma, Burlington, MA, USA) were diluted 1:100 in PBS containing 2% BSA for 2-h incubation at RT. Alexa Fluor 594 goat anti-rabbit IgG (H + L) antibody (A11037; Molecular Probes) and Alexa Fluor 488 goat anti-mouse IgG (H + L) antibody (A11029; Molecular Probes) were also diluted 1:200 in PBS for 40-min incubation at RT. Finally, 4′,6-diamidino-2-phenylindole (DAPI; 10236276001; Roche, Basel, Switzerland) was applied at RT for 10 min, washing with PBS and obtaining photo images by confocal scanning microscope (IX 70; Olympus). Immunofluorescence staining was also performed, using anti-Collagen I (COL-1) antibody (ab34710; Abcam) and Anti-osteocalcin (OCN) antibody (sc-30044; Santa Cruz Biotechnology, Dallas, TX, USA) to detect markers of osteogenic cells within plant scaffolds. Sections of OB-differentiated plant scaffolding were first incubated in 3% H_2_O_2_ to remove endogenous peroxidase activity, and then incubated with 10% normal goat serum containing 1× PBS with 1% BSA (1% PBA) to remove nonspecific background staining. Anti-COL-1 and anti-OCN antibodies were diluted in 5% normal goat serum containing 1% PBA for overnight incubation at 4 °C. Specimens were then incubated with Alexa Fluor 594 goat anti-rabbit IgG (H + L) antibody (A11037; Molecular Probes) in PBS for 40 min at RT. Nuclear staining was achieved using DAPI.

### Animal experiments

To confirm the regenerative capacity of OB-differentiated plant scaffolding in *vivo*, we used male rats (Sprague Dawley) with calvarial defects. The rats were anesthetized using 2% isoflurane, applying ophthalmic liquid gel to protect the eyes. When implanting scaffolds, we used a sterile trapezoidal drill to create 5-mm diameter calvarial defects. The OB-differentiated plant scaffolds were rinsed with sterile PBS prior to placement on defects. We then sutured the skin, administered analgesia, and observed for healing.

### Histological examination of explanted plant scaffolds

Rats with implanted scaffolds were sacrificed by CO_2_ inhalation at 2, 4, and 8 weeks after grafting, fixing the scaffold tissue in 10% formalin. The routinely processed, paraffin-embedded specimens were then sectioned at 5 μm. Masson’s trichrome stain to confirm new bone formation called for tissue fixation in preheated Bouin’s solution (60 min at 60 °C), followed by tap-water rinse to remove the picric acid. After incubation with Weigert’s iron haematoxylin at RT for 10 min and subsequent tap-water rinse, Biebrich scarlet was applied for 5 min at RT. Sections were then rinsed with tap water and incubated in an acid (phosphotungstic + phosphomolybdic) and distilled water solution (1:1:2) for 15 min at RT, followed by 5-min incubation in 2% aniline blue. Sections were ultimately rinsed with tap water and incubated 3 min in 1% acetic acid. Areas of new bone and collagen growth were quantified using ImageJ freeware. To confirm *in vivo* bone regeneration within OB-differentiated apple scaffolding, we used human mitochondrial and osteocalcin antibodies for co-localization staining. Plant scaffold tissue sections were incubated in 3% H_2_O_2_ to remove endogenous peroxidase activity, and then incubated with 10% normal goat serum containing 1% PBA to remove nonspecific background staining. Mouse anti-human mitochondria monoclonal antibody (MAB 1273; Millipore Sigma) and osteocalcin antibody (sc-30044; Santa Cruz Biotechnology) were diluted 1:100 in 5% normal goat serum containing 1% PBA for overnight incubation at 4 °C. Sections were then incubated with Alexa Fluor 594 goat anti-rabbit IgG (H + L) or Alexa Fluor 488 goat anti-mouse IgG (H + L) antibody in PBS for 40 min at RT. Nucleus staining was achieved using DAPI.

### Statistical analysis

All experiments were repeated at least three times, expressing data as mean and standard error of the mean (shown as error bars). All computations and graphic depictions were powered by standard software (Prism 5; GraphPad, San Diego, CA, USA). In comparing three or more groups during non-parametric quantitative data analysis, Kruskal-Wallis and *post hoc* Dunn’s multiple comparison tests were applied. Statistical significance was set at *p* < 0.01 (*p < 0.01; **p < 0.005; and ***p < 0.001).

## Supplementary information

Supplementary Information.
