# Supplementary material for: Induced Osteogenesis in Plants Decellularized Scaffolds
Source: Sci Rep. 2019 Dec 27;9:20194. doi: 10.1038/s41598-019-56651-0 (PMC6934596; doi:10.1038/s41598-019-56651-0)
Supplement: Supplementary file 1 — Supplementary Information. [file 41598_2019_56651_MOESM1_ESM.docx]

**Supplementary Information**

**Induced Osteogenesis in Plants Decellularized Scaffolds**

**Authors:** Jennifer Lee^1*^, Hyerin Jung^2*^, Narae Park^2^, Sung-Hwan Park^1^, Ji Hyeon Ju^1,2^

**Affiliations:**^1^Divison of Rheumatology, Department of Internal Medicine, College of Medicine, Seoul St. Mary's Hospital, The Catholic University of Korea, 222 Banpo-daero, Seocho-gu, Seoul, 06281, Republic of Korea

^2^CiSTEM Laboratory, Convergent Research Consortium for Immunologic Disease, College of Medicine, Seoul St. Mary's Hospital, The Catholic University of Korea, 222 Banpo-daero, Seocho-gu, Seoul, 06591, Republic of Korea

^3^Catholic iPSC Research Center, College of Medicine, The Catholic University of Korea, Seoul, 137-701, Republic of Korea.

*Jennifer Lee and Hyerin Jung equally contributed to this article.

***Correspondence:** juji@catholic.ac.kr

Ji Hyeon Ju MD, PhD, Director, Catholic iPSC Research Center & Professor, Division of Rheumatology, Department of Internal Medicine College of Medicine, Seoul St. Mary's Hospital, The Catholic University of Korea, 222 Banpo-daero, Seocho-gu, Seoul, 06281, Republic of Korea

**Supplementary figure 1**

**
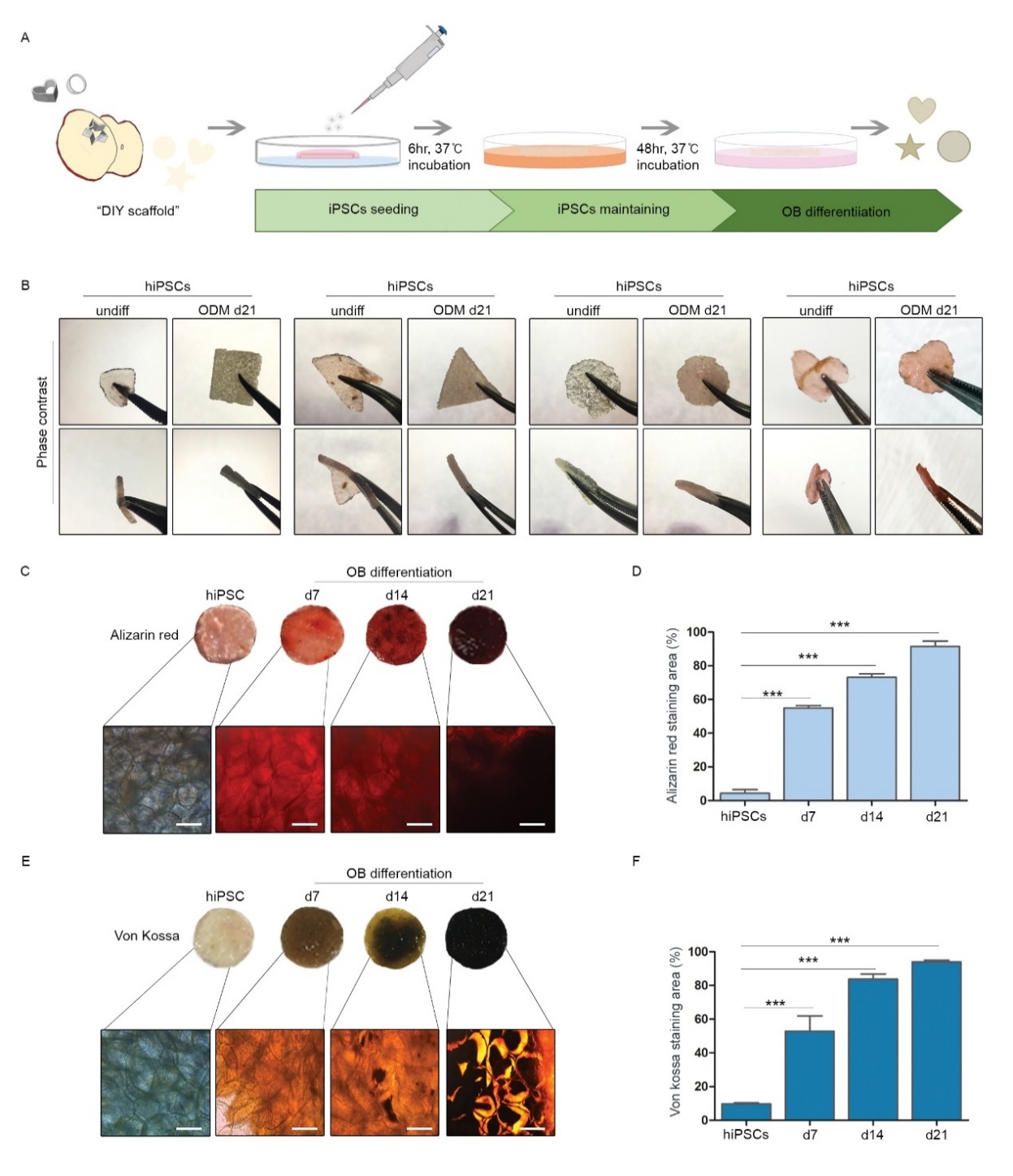
**

**Supplementary Figure S1.** Apple-derived scaffolds can be used for in vitro development of bone-like tissues with various shapes. (A) Experimental scheme. Apple was sliced to various shapes and decellularized. Human induced pluripotent stem cells (hiPSCs) were seeded and differentiated into osteoblasts. (B) Various shapes of scaffolds before (left panels) and after (right panels) osteogenic differentiation. (C-D) Alizarin red staining of the scaffolds according to the number of days after osteoblast differentiation. The graph depicts the proportion of the stained area. (E-F) Von Kossa staining of the scaffolds, according to the days after osteoblast differentiation. The graph depicts the proportion of the stained area.

**Supplementary figure 2**

**
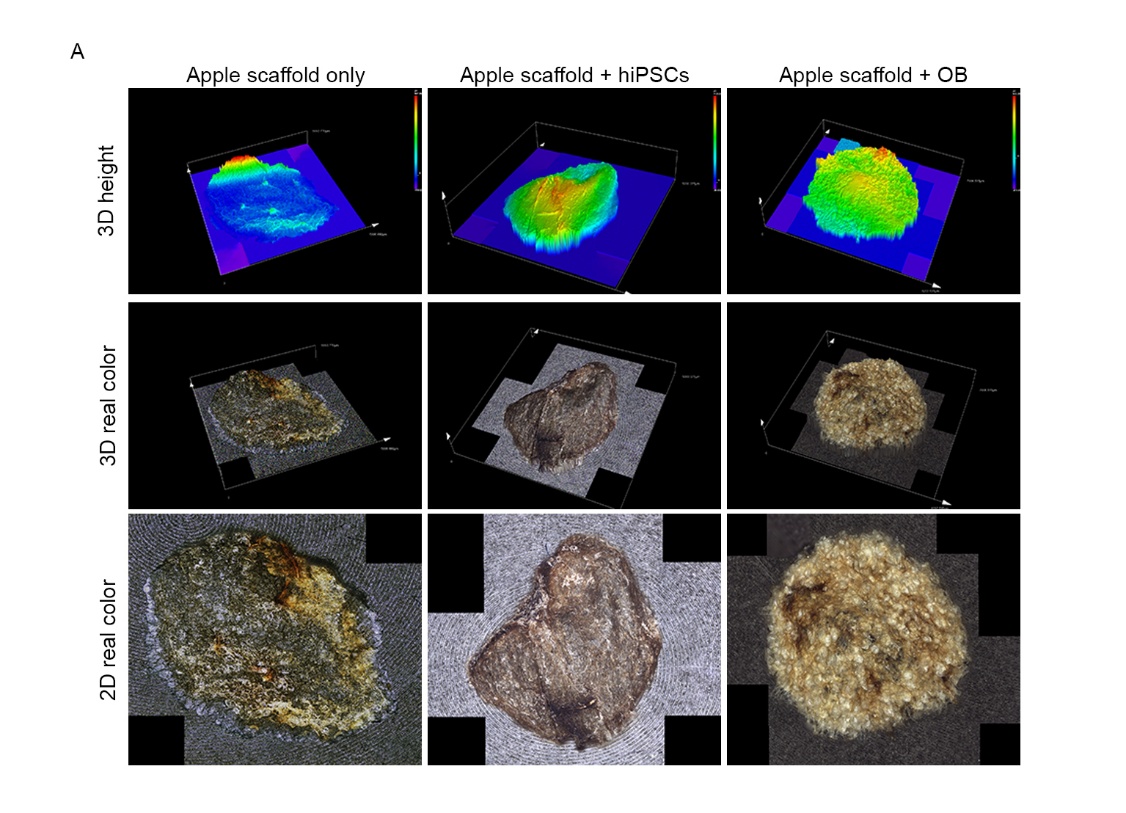
**

**Supplementary Figure S2.** Increase of thickness and surface roughness of layer in osteoblast differentiated apple scaffold. (A) The height of the 3D shape sample was measured and the surface roughness was measured with a laser confocal microscope (LEXT OLS5000, Olympus)

**Supplementary figure 3**

**
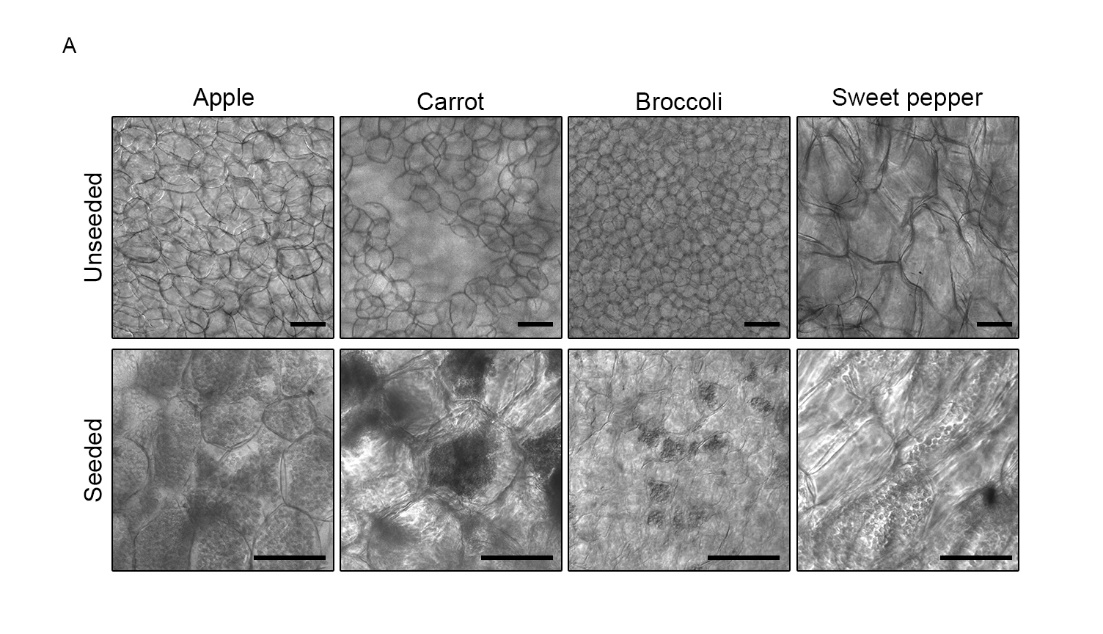
**

**Supplementary Figure S3.** Cell survival and proliferation on Different type scaffolds. (A) In the different type of scaffolds, cells maintained their poorly spread and did not proliferate well.

**Supplementary figure 4**

**
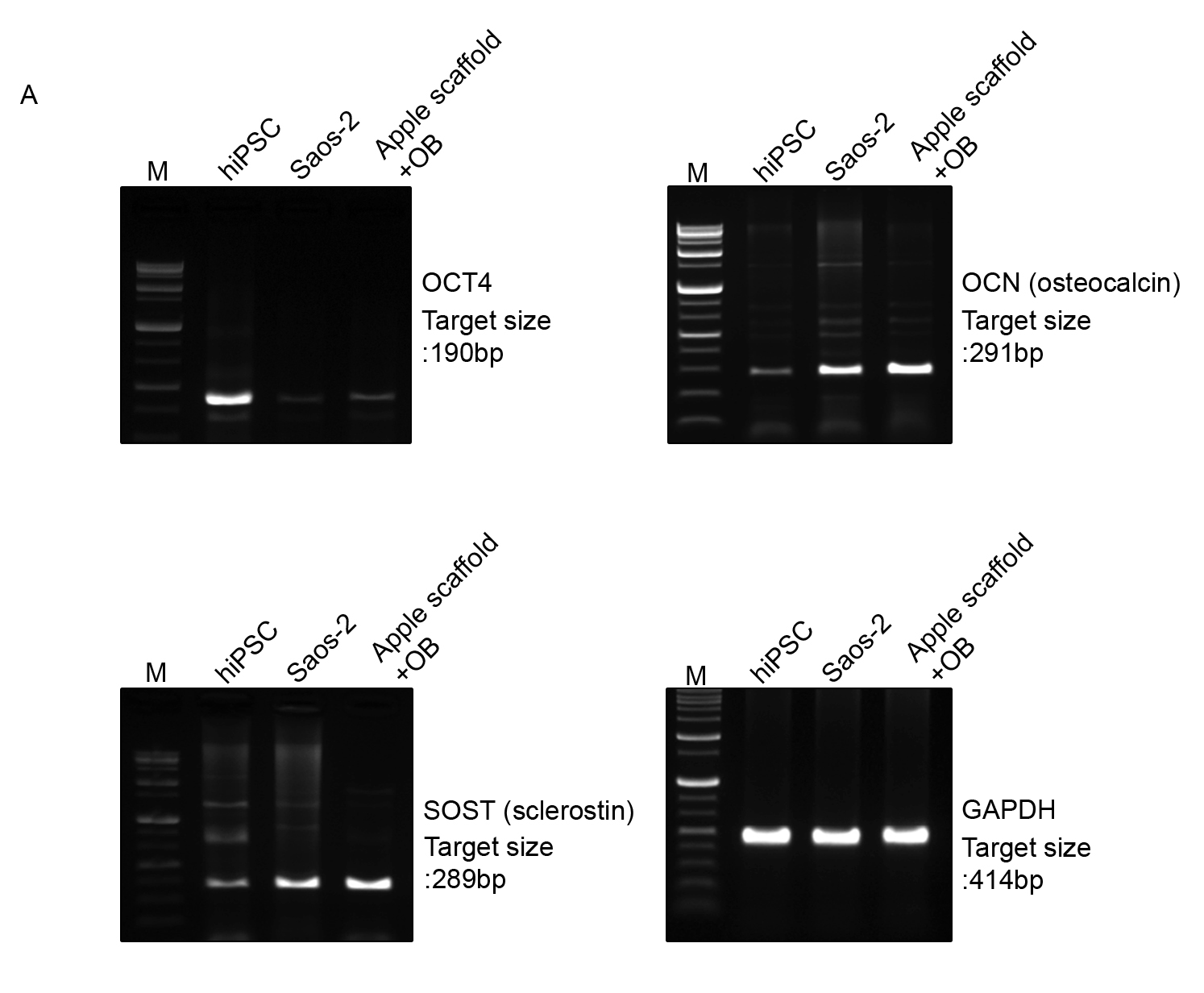
**

**Supplementary Figure S4.** Confirmation of Bone Cell Specific Marker Expression in Apple scaffold + OB. (A) The full-length electrophoretic bands of mRNA expression in hiPSC, Saos-2 and apple scaffold+OB. Gene expression was pluripotent marker of OCT4, late-stage mineralization markers OCN (osteocalcin) and bone cell markers SOST (sclerostin). Saos-2 cells was used as a positive control and GAPDH was used as a housekeeping.
